# Distant metastasis of rectal adenocarcinoma in a temporary tracheostoma

**DOI:** 10.2478/raon-2013-0079

**Published:** 2014-11-05

**Authors:** Robert Sifrer, Primoz Strojan, Nina Zidar, Miha Zargi, Ales Groselj, Milena Krajinovic

**Affiliations:** 1 University Department of Otorhinolaryngology and Head and Neck Surgery, Ljubljana, Slovenia; 2 Institute of Oncology Ljubljana, Ljubljana, Slovenia; 3 Institute of Pathology, Faculty of Medicine, Ljubljana, Slovenia; 4 Department of Otorhinolaryngology, General Hospital Novo mesto, Slovenia

**Keywords:** temporary tracheostoma, distant metastasis, rectal adenocarcinoma

## Abstract

**Background:**

The temporary tracheostoma’s metastases of head and neck cancer had already been reported in the literature. So far, they had been considered as regional dissemination of the malignant disease. We report a case of temporary tracheostoma’s metastasis of carcinoma from non-head-and-neck primary site, what has not been reported in the literature, yet. Therefore, it is the first reported case of the systemic dissemination of malignant tumour into temporary tracheostoma.

**Case report.:**

Fifty-four-year-old female patient, previously treated for a rectal adenocarcinoma, reported in our office with exophytic pink tissue masses around the temporary tracheostoma. The biopsy and immunohistochemistry findings were consistent with temporary tracheostoma’s metastasis of the rectal adenocarcinoma. The patient received palliative radiotherapy and died of systemic progression of the disease.

**Conclusions:**

The patients with history of primary cancer of any origin and exophytic proliferating changes around the tracheostoma require an appropriate diagnostic work-up including a biopsy. The type of treatment depends on the extent of the disease, previous therapy and general condition of the patient.

## Introduction

The occurrence of malignant growths in the region of permanent tracheostoma after laryngectomy is well documented in the literature and is usually considered to be a peristomal recurrence of the primary laryngeal carcinoma. It usually affects patients with squamous cell carcinoma arising in the subglottic larynx, malignant infiltration of the thyroid gland, Delphian or paratracheal lymphatic nodes, those with a pre-resection urgent temporary tracheostomy, or incomplete tumour removal with laryngectomy.^1^,^2^ Therefore, some authors prefer urgent laryngectomy over urgent temporary tracheostomy in case of acute respiratory distress due to a laryngeal tumour.^3^

In comparison to permanent tracheostoma, the impairment of the temporary tracheostoma by malignant disease is rare.^4^ Only individual case reports on metastasis in temporary tracheostomas, originating from head-and-neck primaries, are available. The problem is usually clinically observed a few months after the completion of treatment, when patients are already decannulated. The clinical picture includes proliferating anterior neck masses with respiratory distress, haemoptysis and haemorrhages.^5^,^6^ Background mechanisms include the continuous shedding and seeding of neoplastic cells from the primary tumour with their implantation in the target area.^4^–^7^ In fact, the metastasis in temporary tracheostoma from the upper aerodigestive tract primary carcinoma is a stomal progression of this tumour.

To the best of our knowledge, the metastasis into temporary tracheostoma from a distant primary site has not yet been reported.

## Case report

In January 1999, a 54-year-old female was treated by anterior resection of the rectum with anastomosis for a moderately differentiated rectal adenocarcinoma sited 12 cm above the anocutaneous line. In histopathological examination, a 7 × 4 × 0.9 cm tumour and resection margins free of tumour cells were described. The disease stage was pT3pN0M0, Dukes B. The patient was given a course of 50.4 Gy (in 1.8 Gy daily fractions) of postoperative irradiation using 10 MeV linear accelerator photon beams and the four-field box technique until April 1999.

Adhesiolysis, resection of terminal ileum and end-to-end anastomosis were performed in May 2000 and sigmostomy in December 2003 with no signs of local or regional recurrence.

One week after the third surgery, the patient complained of severe hoarseness but was not examined by an otorhinolaryngologist at that time.

In January 2005, multiple pulmonary metastases were diagnosed and the patient was directed to chemotherapy. By October 2005, she had had seven cycles of FOLFIRI regimen (irinotecan, leucovorin, 5-fluorouracil) resulting in a partial remission. At the time of progression in March 2006, a XELOX regimen (capecitabine, oxaliplatin) was introduced. Due to its ineffectiveness, it was replaced with a FOLFIRI-cetuximab combination after the third cycle in June 2006. By March 2007, the patient had received six applications, also without any clinical benefit. As the patient was still in good performance status, the XELOX chemotherapy was re-started, but was terminated after the forth application in July 2007 due to side effects (allergic reaction with dyspnoea during administration of oxaliplatin).

In August 2007, the patient was admitted to the otolaryngology department with a clinical picture of an acute respiratory distress. During the clinical examination, subglottic stenosis was observed. It was of a concentric type and the mucosa covering it was smooth and showed no sign of malignancy, which was confirmed with direct pharyngolaryngoscopy with rigid tracheoscopy. The stenosis was attributed to an orotracheal intubation injury during the last abdominal surgery. An urgent tracheostomy was performed; after the surgical wound healed and the patency of tracheostoma stabilised, the patient was discharged from the hospital with appropriate knowledge on basic care of her tracheostoma.

**FIGURE 1.**
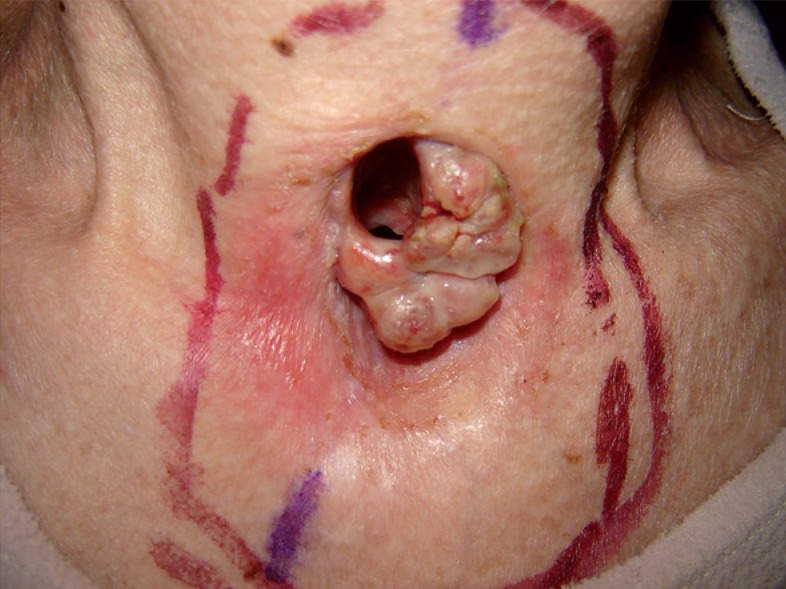
Metastasis of colorectal carcinoma in temporary tracheostoma with margins of irradiation field marked on the skin – anterior view.

In February 2008, difficulties with cannula replacements associated with unusual changes around the tracheostoma prompted the patient to visit the ENT office again. After the removal of the cannula, the clinical examination revealed exophytic pink tissue masses, 1.5 × 1.5 cm in size, growing around the left and inferior rim of the tracheostoma in the area of skin transition into tracheal mucosa (Figure 1), mimicking hypertrophic granulations that are usually found in neglected tracheostomas. Due to extent of these changes, a biopsy was performed.

**FIGURE 2.**
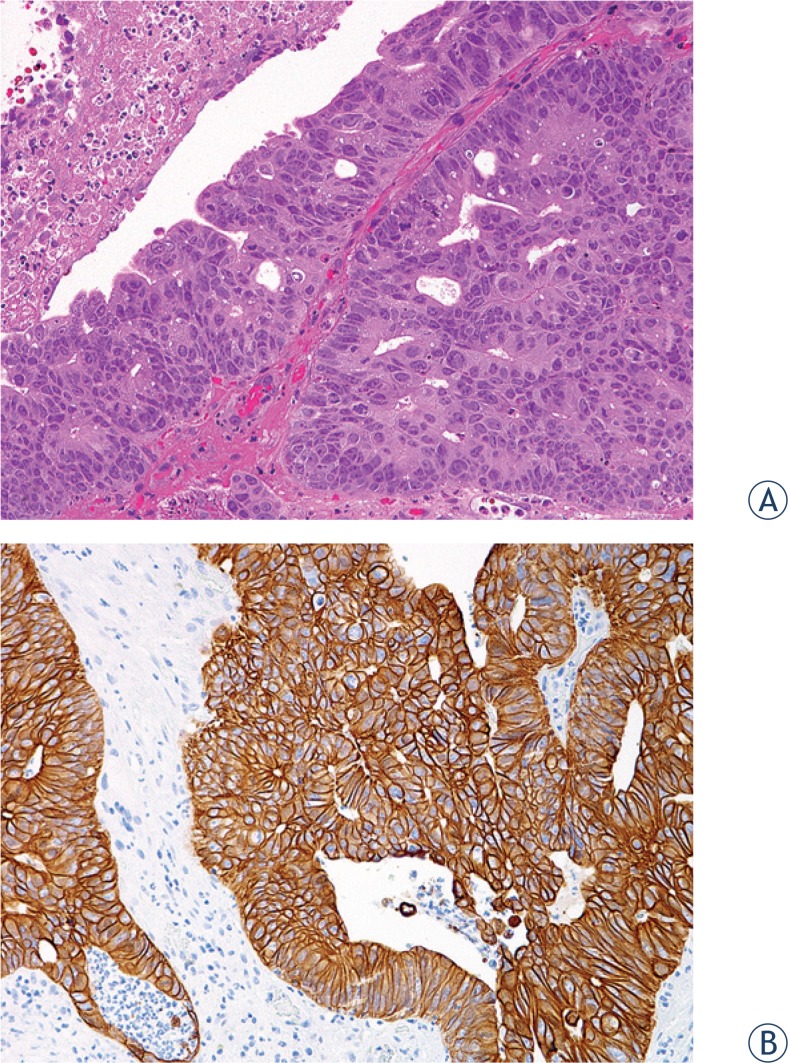
Adenocarcinoma metastatic to tracheostoma. **A.** Atypical tubular glandular structures with abundant necrosis, tumour cells show moderate cellular and nuclear polymorphism. **B.** Immunohistochemical reaction for cytokeratin 20 is strongly positive in tumour cells.

The biopsy sample measured 7 × 5 × 2 mm and consisted of a tumour with abundant necrosis. Histologically, the tumour was composed of atypical glandular tubular structures and islands of tumour cells exhibiting moderate cellular and nuclear pleomorphism, and numerous mitotic figures (Figure 2A). Immunohistochemical analyses showed a diffusely positive reaction for cytokeratin 20 (Figure 2B), and a negative reaction for cytokeratin 7. Morphologic characteristics and the immunophenotypes of the tumour samples from the tracheostoma and the colon were similar, confirming the diagnosis of colon adenocarcinoma metastatic to tracheostoma.

The patient was offered palliative radiotherapy with 10 fractions of 3 Gy/day and an appositional 12 MeV electron beam, covering only the macroscopic disease with margin (Figure 1). At the end of the irradiation course, there was no change in the clinical appearance of the metastasis in the tracheostoma. In May 2008, a CT scan revealed solitary brain metastasis in the left parietal region of 6 cm in diameter with surrounding oedema. She was treated with corticosteroids in addition to whole brain radiotherapy of 20 Gy (4 Gy/day).

The patient died of systemic progression of her malignant disease in June 2008.

## Discussion

In this report, we have described a unique case of distant metastasis from a primary tumour other than the head-and-neck squamous cell carcinoma to temporary tracheostoma. Compared to patients with metastatic disease in tracheostomy sites originating from the head and neck primary tumour, several aspects should be emphasized. Metastasis in tracheostoma from head-and-neck cancer is a loco-regional progression of the disease, whereas in our patient the metastasis in the tracheostoma was the consequence of a systemic dissemination of remote malignant disease with haematogenous route of cancer cells spread as the most probable pathogenetic mechanism.

Colorectal cancer can metastasize even 15 years after treatment of the primary tumour.^8^ In contrast, the incidence of distant metastases in the larynx is low, ranging from 0.09% to 2% of all malignant lesions in this location.^9^,^10^ The preponderant primaries metastasizing to the larynx are cutaneous melanoma, followed by renal cell, breast and lung carcinomas; metastases of colorectal adenocarcinoma account for only 6% of all secondary laryngeal tumours.^11^ The incidence of distant metastases in the trachea is even lower with breast carcinoma, colorectal carcinoma, melanoma, thyroid carcinoma, sarcomas, hepatocellular carcinoma, renal cell carcinomas and esthesioneuroblastoma being reported as origins of metastatic deposits.^12^–^14^ Metastases to the larynx and trachea usually occur *via* a haematogenous route and are seldom lymphogenous.^9^,^11^,^12^,^14^ They appear as endolaryngeal and endotracheal lesions and are considered to be locally advanced diseases with unfavourable prognoses.^11^,^12^

Comparison of histological and immunohistochemical characteristics of primary tumour and the metastasis helps to confirm the diagnosis, as was the case in our patient.^15^ Considering the best treatment option(s) in these patients, one must take into account the extent of the disease in the neck and other body sites (if present), and the patient’s general performance and preference(s). Curative treatment scenarios are limited to patients without simultaneous metastases at other sites and depend on eventual previously established therapies.^9^–^11^,^15^

The newly created temporary tracheostoma is a surgical wound that heals by second intention. Formation of granulation tissue in the wound is usually abundant, creating a fertile bed for seeded neoplastic cells.^5^,^7^,^16^ The latter could be explained by a rich vascular network and lack of inflammatory cells found in these granulations, resulting in a good distribution of nutrients and growth factors, and diminished immune reactivity. Accordingly, we hypothesize that fresh granulations in the recently constructed temporary tracheostoma provided a fertile soil, although at an unusual site, for haematogenously metastasizing neoplastic cells of colorectal adenocarcinoma.

## Conclusions

Our case clearly shows that in patients with temporary tracheostoma and history of primary cancer of any origin, including below the clavicles, careful follow-up is required. When exophytic proliferating masses around the tracheostoma are observed, a biopsy is mandatory to exclude malignancy. Unless other distant metastases had already been confirmed, an appropriate diagnostic work-up should be done. If temporary tracheostoma is the only site of metastatic disease, the intent of therapy should be curative; otherwise only palliative measures are indicated. The curative treatment can be surgical, consisting of resection of metastasis with extended laryngectomy, or radiotherapy with the inclusion of visible metastasis and potential sites of eventual microscopic disease in high-dose irradiation volumes. The type of primary treatment depends on the extent of the disease, previous therapy (if any) and the general condition and preferences of the patient.
